# Factors Influencing Co‐Use of Tobacco and Cannabis Amongst Young Adults in UK Further Education Colleges: A Qualitative Study

**DOI:** 10.1111/dar.70002

**Published:** 2025-06-24

**Authors:** Hannah Walsh, Ann McNeill, Maria Duaso

**Affiliations:** ^1^ Florence Nightingale Faculty of Nursing, Midwifery and Neuroscience King's College London London UK; ^2^ Department of Addictions, Institute of Psychiatry, Psychology and Neuroscience King's College London London UK

**Keywords:** cannabis, co‐use, mental health, tobacco, young adults

## Abstract

**Introduction:**

Tobacco and cannabis are commonly co‐used (i.e., used concurrently or co‐administered) but rarely ‘co‐addressed’ and few co‐use interventions exist. Young adulthood presents a key age for intervening in substance use, therefore understanding young adults' perspective is crucial, but little is known about how they understand or experience co‐use. This study aimed to explore factors influencing co‐use and changes to use within a population of young adults in the UK.

**Methods:**

Participants were young adults recruited via three Further Education (vocational) colleges, who reported past 6‐month co‐use. Individual semi‐structured interviews were carried out and analysed using the Framework approach.

**Results:**

Eighteen participants were interviewed. Analysis identified influential factors, comprising three categories: (i) identity and social context including the concept of co‐use; (ii) experiences; and (iii) understanding of effects. Family and peers were an important influence on use and cessation and young adults used their observation of peers' experiences to understand potential harms of each substance, as well as the complex relationship between cannabis use and mental health.

**Discussion and Conclusions:**

A broad range of factors influence co‐use, and changes made to co‐use, of tobacco and cannabis in young adults. Further investigation is needed to inform the development of co‐use interventions. Credible co‐use health risk information relevant to young adults, and the role of co‐occurring mental health challenges need consideration in the development of co‐use interventions.

## 1 Introduction

Tobacco and cannabis are two of the most commonly used substances worldwide and are often consumed together. The term *co‐use* is used to refer to any use of tobacco and cannabis, whether co‐administered or concurrent. Co‐administration describes the use of both substances in the same product, that is, a joint, or spliff; and concurrent use describes any use of tobacco and any use of cannabis within a given time period [1]. The time period for concurrent use may be defined as ‘same occasion,’ or ‘same day’, or over a number of days [2]. It is recommended that surveys measure co‐use over at least the past 12 months and where possible to also include past month use both to capture a broader range and variability in patterns of co‐use [3].

Cannabis routes of administration differ worldwide. In Europe and Australasia, cannabis is predominantly used with tobacco, that is, co‐administered, whereas in North America tobacco and cannabis are typically used concurrently [4]. Co‐administration has been described as having a partly practical purpose as it facilitates a ‘dilution’ of cannabis and therefore allows greater control over cannabis consumption [5, 6]. Reasons for concurrent use might include tobacco used immediately following cannabis to enhance or prolong the cannabis effects [2].

Despite co‐use being so common, ‘co‐treatment’ (e.g., interventions which address both tobacco and cannabis together) is not. Few interventions which address co‐use have been fully tested, and most interventions which address one substance typically do not assess or address co‐use of the other substance [7, 8].

Worldwide, an estimated 22% of the population used tobacco in 2020, and 4% used cannabis [9]. In the United Kingdom, tobacco smoking prevalence was 14.4% in 2019, though amongst those aged 16–24, it was 21% [10]. UK prevalence of past year cannabis use was estimated to be 7.8% in 2020, and also higher amongst those aged 16–24 at 18.7% [11]. US studies suggest that despite tobacco smoking prevalence decreasing in the whole population, it is increasing amongst adult cannabis users, with an estimated 54% of daily cannabis users also smoking tobacco [12]. Similarly, amongst US daily tobacco smokers over the age of 12, 9.0% report smoking cannabis daily [13]. Co‐use of tobacco and cannabis is described in UK studies, with 21.9% of people who smoke tobacco reporting past year cannabis use and 43.3% of people who use cannabis reporting smoking tobacco [14]. That study also identified a ‘hidden cohort’ of people who co‐use, as over half of the participants who reported cannabis use also indicated they did *not* smoke tobacco, nevertheless administered cannabis with tobacco.

Qualitative literature expands our understanding of the function of co‐use. A study of Canadian adolescents identified differences in the health beliefs and risk perceptions of the two substances where tobacco was largely seen as harmful to health, but cannabis was considered a ‘natural’ product and therefore less harmful [15]. Young adult cannabis and co‐users in Switzerland also perceived cannabis as less harmful than tobacco and described an ambivalent boundary between dependence on tobacco versus cannabis and noted the potential to develop tobacco dependence after a period of co‐use [16]. UK studies describe the role of cannabis in maintaining tobacco use and/or dependence, and that the two are intricately related [5, 6, 17]. In Amos's study, participants described how quitting tobacco was difficult when they still needed it to facilitate their cannabis use.

Co‐use elicits concern as it may pose harms beyond those of each substance in isolation. For example, respiratory symptoms appear worse amongst those who co‐use compared to smoking tobacco only [18], the potential for cannabis dependence may increase and impact smoking cessation outcomes [19] and some forms of co‐use may increase carbon monoxide exposure when compared to single substance use [20]. There is a strong positive relationship amongst adolescents and young adults between co‐use and mental health conditions, along with an increase in risk behaviours and neurocognitive consequences [21]. Peters et al. found a higher rate of psychosocial problems amongst adults who co‐used in comparison to those who used cannabis only, but not in comparison with those who used only tobacco [22].

Given the frequency of co‐use, the interaction between tobacco and cannabis use and treatment demand for cannabis use disorder [23, 24], there is a clear need for effective co‐use interventions [8, 25]. Young adulthood is a key age range to intervene in substance use trajectories, to prevent use becoming regular or dependent which may negatively impact psychological and physical wellbeing. Understanding how changes in co‐use practices occur with and without intervention, and what factors influence such changes, could inform the development of targeted interventions addressing co‐use within this age group. Further, the extent to which ‘co‐use’ as a concept is understood, or relevant for people who use both tobacco and cannabis, is not known.

This study therefore aimed to: (i) identify factors which influence use and understanding of tobacco and cannabis co‐use in young adults; and (ii) explore the experiences and attitudes of people who co‐use towards changing use of either or both.

## 2 Methods

### 2.1 Participants and Setting

Young adults were recruited through a linked cross‐sectional survey exploring tobacco and cannabis co‐use (*n* = 141) [26]. Students aged between 16 and 30 who reported past six‐month use of both tobacco and cannabis were eligible for the survey. A six‐month period was chosen in anticipation that use status during this age is likely to change often, and as the study aimed to explore changes to use including interest and lack of interest in cessation or reduction, then both those who used at the time of the survey or had recently quit were eligible. Although co‐use measurement recommendations suggest a minimum timeframe of 12 months, 6 months seemed reasonable to capture changes in use while balancing the potential for recall bias [3]. Although this could theoretically include people who had used both substances at different time periods, it was anticipated that most of the cannabis use would be co‐administered with tobacco as is typical for the UK.

The survey recruited students attending UK Further Education (FE) Colleges which deliver numeracy, literacy, vocational and some degree‐level courses [27]. These settings were chosen to capture the experiences of students from a diverse range of socio‐economic backgrounds and educational achievement.

### 2.2 Recruitment

Respondents to the survey were invited to leave details if they were interested in participating in an interview (*n* = 111). A sequential sampling process was used to maximise the inclusion of interviewees who had experience of a quit attempt, given the study aims. The 111 students were sorted into one of three groups: successfully quit both, made a quit attempt of either, or made no attempt to change either. Participants from the first group were contacted by text message, and those responding then received a short call to discuss the interview process and were offered either in‐person or phone interviews. Once all respondents from the first group had been contacted and interviewees recruited, the second then third group were contacted.

### 2.3 Sample Size

The concept of ‘information power’ as an alternative to data saturation in qualitative research was considered. This uses five criteria to determine the amount of ‘information power’ a sample holds including: breadth of aim, specificity of target sample, use of established theory, quality of interview dialogue and the use of case or cross analysis [28]. After each interview, the cumulative dataset was reviewed with the supervisory team, using the concept of information power. Considering the focused aim, use of theory, the specificity of the target sample, the richness of the dialogue and breadth of sample characteristics, it was agreed after the 18th interview the dataset was sufficient for meaningful analysis.

### 2.4 Ethical Approval

Ethical approval was sought from a university committee, reference HR‐17/18‐7583. Each participant was offered a £25 shopping voucher to thank them for their participation. At the start of each interview, the researcher sought formal consent, discussed confidentiality and study withdrawal, and answered any questions.

### 2.5 Data Collection

Interviews took place between February and December 2019 and were conducted by the lead author (HW). These ranged in duration from 21 min to 1 h. Each interview was recorded, and the first four were transcribed by HW, as a way of reviewing the interview process and becoming familiar with the data at an early stage [29]. Subsequent interviews were transcribed by a transcription company under a confidentiality agreement with the university. Recordings and transcriptions were then stored securely online, as per university guidance.

The semi‐structured interview questions covered the following topics:

- Current tobacco, cannabis and co‐use;
- Experience and attitudes towards changing tobacco and/or cannabis use;
- Context and environmental factors which could influence use or changing use;
- Motivation and expectations about changing use.

Care was taken to ensure that the same questions were asked equally of tobacco and cannabis, to reveal salient aspects of each substance through comparison.

### 2.6 Data Analysis

The framework approach was selected as means of organising the data and supporting the transition from descriptive to inductive findings, and NVivo (2020) was used to organise the analysis process. The framework approach allows for analysis to move beyond description to conceptual explanations and supports a transparent process of tracking the raw data elements to the final outcomes [30, 31]. Analysis comprised five stages; familiarisation, indexing, identifying a framework, charting and mapping [32]. Familiarisation included close re‐reading of the transcripts and initial notes made. Indexing comprised assigning ‘codes’ and took place in conjunction with the framework creation, the third stage. An initial thematic framework was developed which reflected the interview schedule structure, and used to code the first transcript by both HW and MD. They then discussed the ‘fit’ of the framework and agreed it did not fully capture all the data, so a second transcript was coded by both authors using an ‘open’ coding process instead. A second framework was then created from this comparative process. Once completed, this version was discussed with all three authors, and H.W. subsequently coded all remaining transcripts using it. The fourth stage comprised charting the data; creating a matrix in NVivo for each of the seven sub‐categories within the framework, and ‘charting’ what each person, (or ‘case’), had said in relation to each of the categories; this was then summarised. The final stage, mapping, involved condensing the summaries further, to ‘distil’ the meaning within each category, and move beyond description to interpretive concepts. The concepts were then clustered according to connective themes, and the final structure of the data realised. The themes were discussed with MD and AM to ensure validity.

### 2.7 Researcher Reflexivity

The lead author (HW) made notes of their experiences, impressions and initial thoughts following each interview, which formed the basis for reflection with co‐authors on responses, positionality and approach for subsequent interviews, including establishing rapport and trust with interviewees and consideration of the credibility of the interview process. This process was intended to acknowledge and respond to potential biases in both the interview and analysis process and allow for implicit and explicit influences to be identified.

## 3 Results

Eighteen students were interviewed. The average age of participants was 18, and ages ranged from 17 to 24. The majority were white British (72%) and female (55%). Co‐use at the time of interview varied, but all had previously co‐administered tobacco and cannabis. See Table 1 for participant characteristics.

**TABLE 1:** Participant characteristics.

Pseudonym	Gender	Age	Ethnicity	Current co‐use	Quit attempt history
Catrina	Female	18	White Irish	Co‐administration. No additional cigarette use	None
Damian	Male	19	White British	Tobacco cigarettes only	Quit cannabis, not tried to quit tobacco
Danielle	Female	20	White British	Tobacco cigarettes only	Quit cannabis, tried to quit tobacco multiple times
Demetrios	Male	17	White European	Co‐administration. No additional cigarette use	Previous episodes of abstinence from both
Efua	Female	17	Black African	Co‐administration and additional tobacco cigarette use	Previous episodes of abstinence from both
Gemma	Female	17	White British	Cannabis use without any tobacco	Reduced cannabis use frequency, stopped tobacco
Imani	Female	21	Black African	Co‐administration and additional tobacco cigarette use	Previous episodes of abstinence from both
Ivan	Male	17	White British	Co‐administration and additional tobacco cigarette use	Tried to quit tobacco
Jamie	Male	24	White British	Co‐administration and additional tobacco cigarette use	Reduced both
Jason	Male	20	White British	Co‐administration and additional tobacco cigarette use	Previously stopped cannabis for 2 months
Jemila	Female	18	Black African	Stopped both	
Kelly	Female	17	White British	Co‐administration. No additional cigarette use	Previously stopped tobacco without cannabis
Laura	Female	17	White British	Co‐administration and additional tobacco cigarette use.	None
Lewis	Male	17	White	Co‐administration and additional tobacco cigarette use	None
Marius	Male	19	White European	Co‐administration and additional tobacco cigarette use	Quit tobacco in past, occasional cannabis breaks
Miriam	Female	17	Asian + Central American	Cannabis use, no tobacco use	Stopped tobacco
Owen	Male	17	White British	Stopped both	
Sinead	Female	17	White British	Co‐administration and additional tobacco cigarette use	One period of tobacco abstinence

### 3.1 Thematic Framework Structure

Interpretive findings relating to the theme of ‘influences’ were categorised further into three sub‐themes: (i) Identity and social context; (ii) Experiences of using tobacco and cannabis; and (iii) Understanding of the effects and impact of co‐use.

### 3.2 Identity and Social Context of Co‐Use

#### 3.2.1 Concept of ‘Co‐Use’

Co‐administration was considered the ‘default’ route of cannabis consumption, since rolling papers and tobacco were the most readily available and cheapest option compared to using other devices such as bongs or vapourisers, and tobacco would facilitate burning of even a minimal quantity of cannabis. Co‐administration was also described as the most sociable, since a joint can be shared, and the process of rolling a joint was a skill to be learned and validated by peers. The addition of tobacco to a joint was also referenced as a means of harm reduction, for example, a way to consume less cannabis, and the amount of a tobacco in a joint was described as:

'a chip, not a full fag' (Demetrios).

The contrast between the use of tobacco in a joint, but dislike of smoking cigarettes was also noted:

'when I started smoking [cannabis] again in the back of my head there was a thought of was it maybe the nicotine in the tobacco that made me want to go back to it?'

Interviewer: 'Do you also smoke cigarettes?'

'No, never. To me that's disgusting' (Lewis).

This suggests that the tobacco in a joint is considered differently to the tobacco from a cigarette, and there is a reluctance to acknowledge they are in fact the same substance, and to identify as a ‘tobacco smoker’ in preference to being a ‘cannabis smoker’.

Although participants described the practice of co‐use, they did not fully acknowledge the implications, nor readily identify the concept.

#### 3.2.2 Family

Participants could be first introduced to tobacco or cannabis through a family member; for example, a mother who smoked cannabis for pain relief. Where parents smoked either tobacco or cannabis, this created a pro‐smoking environment. In some cases, mothers purchased tobacco for them, and others smoked with their mother, as it brought them together:

'If it was less available then I would smoke less, it's as simple as that … my parents smoke obviously as well, so it's just one of them things, where it's quite obvious how it's played out.' (Lewis).

Conversely, some participants said their family tolerated but did not support their cannabis use, and interviewees who were not open about their use, or the extent of their use, reported being uncomfortable about being dishonest with their family.

Witnessing a parent stopping smoking created a shift in perspective, reinforcing the possibility of change:

'I think it does help if you come from a home where others are like smokers, then like, your parents, like the adults lead by example. I think when, you know, you have someone that you can look up to, a friend of yours that's stopped' (Imani).

#### 3.2.3 Friends, Peers and Relationships

Friends, peer groups and relationships were key to enabling initiation and ongoing access to both tobacco and cannabis, and in setting the norms for use. The ubiquity of cannabis was apparent, where friends who did not use cannabis were the exception, and there was usually someone nearby you could smoke cannabis with:

'I started doing it just socially because my friends around me were doing it, so I was doing it as well. I wouldn't ever buy it'(Gemma).

Smoking cannabis was sometimes considered a feature of a particular ‘scene’, meaning participants could feel that if they were to quit, they would need to develop new friendships outside of that scene or hide the fact that they had stopped.

For others, the support of their friends was seen as key to prompting abstinence or reducing their use:

'… I think mostly I'd just need the support of my friends. Like “I could just smoke this one”, if they were like “but you're trying to quit” then I'd say you're right. Let me back up that thought' (Catrina).

Some participants had been introduced to cannabis by their partners, which provided easy availability, especially if they were a dealer. However, when partners held negative opinions of smoking tobacco or cannabis, it sometimes prompted consideration of reducing or stopping their use.

#### 3.2.4 Culture

Specific examples of cultural attitudes towards tobacco and cannabis were described as influencing use:

'Well in the Black community or more like my age kind of Black people, smoking cigarettes … it's kind of frowned upon. It's like “eurgh, why are you smoking cigarettes when you could smoke a joint?”’ (Imani).

For one participant, this perception that Black culture included cannabis use meant that their Black boyfriend was frequently stopped in their car by the police and searched for drugs. This persistent racial profiling eventually led him to stop using cannabis.

#### 3.2.5 Workplace

A pro‐tobacco culture in the workplace had a substantial impact on whether participants smoked. One described his manager addressing a group of new apprentices:

‘“How many of you smoke?”’ Hands up. “How many of you don't smoke? How many of you quit?” … The ones that don't smoke, give it two weeks, you will. Same with the ones that quit smoking. The ones that do smoke, you'll be smoking a hell of a lot more' (Damian).

In a stressful environment, smoking tobacco, and/or cannabis together after work was for unwinding, whereas when work was unrewarding or repetitive, cannabis would provide a respite from boredom. While moving on to more meaningful work sometimes decreased use, a greater available income could also increase use. Some participants were regularly urine drug tested at work (e.g., trainee rail drivers), which led to them being more careful about when they would use cannabis.

### 3.3 Experiences of Use

#### 3.3.1 Mental Health

The relationship described between cannabis use and mental health was complex, multi‐faceted and multi‐directional; whereas the role of tobacco in mental health was only occasionally referenced in relation to using tobacco alongside experiencing stress or distress.

Some participants felt that cannabis was a useful tool for managing their mental health by acting as a buffer between the self and difficult emotions and allowing them to ‘escape’ into their own world, and to manage anxiety or low mood.

'I think it's like an escape for some people, when real life gets too much for them. It's a bit like sleeping but you still function'(Miriam).

While cannabis made some anxious participants feel more relaxed, it could also trigger anxiety through physical experiences such as increased alertness and a faster heartbeat:

'… It's kind of like when I use it I have two conflicting feelings, so on one hand I feel really chilled out and like I'm zoning out and what not, but on the other hand, the anxious bit is also there, like it's present' (Efua).

While cannabis use was perceived as providing protection during difficult life experiences, it could also exacerbate negative thinking, and indeed both effects might be experienced by an individual simultaneously. Participants believed that cannabis use sometimes caused the onset of depression or even suicidal thoughts, and psychosis was noted as a potential outcome of cannabis use, although others were sceptical about this link. Some had chosen to delay cannabis use until they were older due to concern over their mental health, others noted an increased risk amongst people with pre‐existing mental health conditions.

'… some of them [friends] had quite bad depression, … and I feel like sometimes it can have both a positive and a negative effect' (Jamie).

#### 3.3.2 Heightened Perceptions and Altered Mood

Cannabis use was described as heightening both senses and experiences. Some chose to use cannabis because they experienced beneficial effects on thinking processes, focus and concentration on their studies;

'It keeps you focussed on one thing, and literally just get it done. Because there's an option for you to block stuff out … it just gets done quicker' (Miriam).

Equally, the intense concentration could lead to overthinking and retreating into yourself, so to avoiding cannabis use when alone was a means of managing this.

Used socially, cannabis could help to facilitate interactions with peers, provide an opportunity to meet others with a similar lifestyle and values, and promote confidence, for example, with someone you found attractive. However, using cannabis in a negative social atmosphere could heighten emotions that could lead to social withdrawal.

Negative aspects of heightened perception were described. For example, hallucinations and paranoia could linger for days after using cannabis, such as a fear of people judging you:

'… it makes me very anxious as well—like if someone's looking at you too long you think oh my god are they looking at me because I look a certain way or … ‐you get very paranoid' (Imani).

Such experiences were an indicator for some that they should stop or reduce their cannabis use.

### 3.4 Understanding of Impacts

#### 3.4.1 Seeking Information

Differing approaches to gathering information on the impact and harms of cannabis compared to tobacco use were described.

For cannabis, the range of approaches included trusting the description of effects provided by their dealer, whereas others attempted to find information from ‘official’ sources, such as National Health Service websites. When they did not find any turned to informal advice elsewhere online, such as ‘Reddit’:

'I was just on there one day and there was like a whole little sub‐reddit dedicated to it [cannabis vapourising]. So I was kind of like, “Okay, what's this all about?” Then I just found a link and ordered one' (Jamie).

By contrast, participants often referenced the information regarding the impact of tobacco they had been given in schools and this, plus risks communicated by health organisations, were largely accepted as fact.

#### 3.4.2 Credibility of Information

The credibility of sources of information appeared important, and balance was an indicator of this. Unlikely or extreme statements about risks were quickly dismissed. National Health Service information sites were noted to provide little acknowledgement of the positive aspects of use and so were considered biased and therefore less credible.

#### 3.4.3 Knowledge Based on Experience

The construction of knowledge regarding tobacco and cannabis contrasted distinctly. Understanding of tobacco and potential harms was consistent amongst participants. By contrast, cannabis knowledge was very varied, with participants building this knowledge base from multiple sources. Unlike their knowledge of tobacco, which was dominated by public health information provided at school and college, participants described using a combination of ‘common knowledge,’ personal experience and observation of others to form conclusions about cannabis use:

'It's not like I sit in a school and get taught about weed. If I did, then that would be great. But you've got to learn it yourself' (Demetrios).

When understanding the effects and risks of cannabis use, their own experiences and those of trusted dealers were highly regarded:

'I trust my shotters [dealers] that I know, because they have knowledge, but if they're some random guy I don't know, then obviously that's different' (Demetrios).

Those participants who had not seen obvious consequence of cannabis use in their own lives tended to dismiss information about the potential negative effects.

Personal experience, opinion and witnessed experiences of peers was a considerable influence on cannabis use and views of potential cannabis health harms. This contrasted with views on the health harms of tobacco. Since participants had learned about tobacco health risks at school, and the public health messaging describing harms had been consistently referenced during their lifetimes, they appeared more likely to accept these harms as fact, without questioning the credibility of these.

## 4 Discussion

To our knowledge, this study is the first qualitative exploration of how young adults who predominantly co‐administer tobacco and cannabis in the United Kingdom describe factors influencing choices and cessation or reduction of each substance. The study found that despite co‐administration being common, young adults identified more readily as cannabis users and tended to view the tobacco in their joints differently from that in a cigarette. They described how family, friends and peers shaped their use and changes to use of each substance, through setting social norms and enabling initiation and ongoing use. Cannabis, in particular, was described as ubiquitous within their circles of college, work and social life. Experiences of poor mental health and heightened perceptions were linked to cannabis use but not to tobacco. A contrast was noted between the availability and credibility of information regarding the impact of tobacco in comparison to that of cannabis.

While previous literature has examined the role of context and culture on young adult tobacco use behaviours [33, 34], there is comparatively less evidence relating to the role within cannabis use from regions where co‐administration dominates [35]. The importance of the socio‐cultural context is identified elsewhere as a major influence on cannabis use within university students in Spain [36], and familial influences in Irish treatment seeking individuals [37]. However, we need to understand more about how a broader range of contexts and lived experience might shape use and changes made to use. For example, although our study included a small number of Black participants, importantly they highlighted how cannabis smoking was distinctly favoured over tobacco in their communities. Choices were also made around cannabis use given the increased risk that Black students faced of being searched by the police. While some findings from the current study have been explored elsewhere [38, 39] further research specifically with Black communities on this topic is needed.

The perceived relationship between mental health and cannabis use was found to be important. Cannabis appeared to act as both a source and salve of mental health problems. This ‘dual’ role of cannabis is identified elsewhere [40] including the use to manage difficult emotional states [41], and evidence for positive and negative relationships between cannabis and psychosis [42]. Notably far fewer associations made between psychological functioning and tobacco use, despite prior evidence of the significance of that relationship [43, 44].

Without information relating to cannabis harms which is perceived as credible, our study confirmed that young adults use contextual information, such as their own and peers' experiences to inform their choices, and particularly their ‘harm reduction’ practices. Credibility was perceived as balanced information which include the positive impact of cannabis, findings which are supported by a recent Spanish study showed that young people choose to omit adverse outcomes and deny risks outlined in the scientific literature [45], and another study which identified that young adults sought information which did not focus solely on harms of cannabis use [46]. These findings together suggest that credibility is a key criterion for young adults when evaluating information, and that this needs to be taken into account when developing health‐related materials aimed at this age group.

### 4.1 Strengths and Limitations

Recruitment from FE colleges instead of universities has provided findings from a population rarely represented in research [47]. However, recruiting in a single region does limit the generalisability of the findings to this location and to the practice of co‐administration. Future research should focus on exploring the importance of different socio‐cultural contexts in which young people may co‐use tobacco and cannabis to ensure the development of culturally informed interventions.

Although the interview schedule balanced questions about tobacco and cannabis equally, it was notable that participants talked at greater length about cannabis. This is reflected in the finding that knowledge and opinion regarding cannabis was far more varied in scope, whereas knowledge and understanding of tobacco tended to centre on ‘accepted’ evidence. In future intervention development, it would be important to recognise that when co‐use is discussed, and despite the fact that most cannabis use in the United Kingdom is in fact co‐use with tobacco, it is cannabis that usually dominates, perhaps because identity as a cannabis user seems more acceptable than identity as a tobacco smoker [33]. It would be therefore important to ensure that both substances are assessed and addressed in intervention content.

Lastly, the research interviews were carried out prior to the COVID‐19 pandemic and thus do not reflect the increase in the prevalence of youth vaping, or changes to cannabis consumption methods in an evolving marketplace. However, the use of tobacco as a ‘carrier’ for cannabis smoking is likely to remain dominant for practical reasons and amongst populations without the means or resources for alternative routes of administration, and thus these findings are expected to retain value over time.

### 4.2 Implications for Research and Practice

Current policy and practice in the United Kingdom regarding tobacco and cannabis is usually led and delivered by separate organisations, one pathway focussing on tobacco dependence treatment, another pathway delivering cannabis use treatment services within substance misuse services. In most instances, these services operate separately, in contrast to the common practice of people who co‐use. The findings within this study support previous calls for co‐use interventions [25], but adaptations of single substance interventions for tobacco and cannabis use which take into account co‐use are also warranted. Policy development is required which could better align or combine these two treatment pathways. Any such interventions need to consider the role that culture and social norms play in both tobacco and cannabis use.

Lastly, harm reduction advice and signposting for people who co‐use but may not seek support are needed. The dearth of accessible, credible, critically reviewed information on cannabis use, risks and harms for young people contrasts with participants' understanding of tobacco‐related risks and harms. Until resources are harnessed to address this challenge, young adults may continue harm reduction based on erroneous beliefs derived from potentially unreliable sources, such as online discussion fora. In collaboration with young adults, policy‐makers, educators, clinicians and researchers should be encouraged to develop such resources, making use of the motivation expressed for harm reduction to prevent problematic use of either substance [48, 49].

## 5 Conclusions

Factors including identity, culture, mental health, family and peers' experiences influence young adult co‐use and changes made to co‐use patterns. Further, attitudes towards changing tobacco and cannabis use are impacted by their understanding of the impact of co‐use and by the variance in credible, accessible information regarding potential health harms of cannabis in comparison to tobacco.

## Author Contributions

Each author certifies that their contribution to this work meets the standards of the International Committee of Medical Journal Editors.

## Ethics Statement

All procedures followed were in accordance with the ethical standards of the responsible committee on human experimentation (institutional and national) and with the Helsinki Declaration of 1975, as revised in 2000 [5]. Informed consent was obtained from all patients for being included in the study.

## Conflicts of Interest

The authors declare no conflicts of interest.

## Data Availability

Original data can be provided on request.
